# Risk factors for college students’ online lending between different genders-A cross-sectional study in China

**DOI:** 10.3389/fpsyg.2023.965049

**Published:** 2023-01-17

**Authors:** Yan Zhang, Lun Luo, Pan Li, Yun Xu, Zi Chen

**Affiliations:** ^1^Chengdu Second People’s Hospital, Chengdu, Sichuan, China; ^2^Chengdu Medical College, Chengdu, Sichuan, China

**Keywords:** online lending, risk factors, gender difference, medical college students, intervention strategies

## Abstract

**Background:**

Online lending on campus is given more attention by researchers as its prominent adverse effects on students. The deficiencies of the previous studies on its psychological factors and intervention strategies were only based on qualitative research. Moreover, there is no study on gender differences. Therefore, our study aims to analyze the gender differences in psychological risk factors and give some practical suggestions for the intervention by quantitative methods.

**Method:**

This is a cross-sectional survey among medical college students in Chengdu. A total of 984 effective questionnaires were collected. The questionnaire includes demographic data, monthly expenses, self-evaluation for three central psychology causing online lending based on empiricism (conformity, comparison, and hedonism), and three psychological assessment instruments (the Chinese version of the Satisfaction with Life Scale, Egna Minnen av Barndoms Uppfostran, and 144-item version of Temperament and Character Inventory). *T*-test*/χ*^2^-test and Binary logistic regression were used to analyze the gender differences in variables and the risk factors of online lending for males and females, respectively.

**Results:**

The utilization rate of online lending exhibited a significant gender difference (*p* < 0.001). In addition, there were gender differences in the scores on SWLS and some subscales of C-EMBU and TCI-144. The risk factors for males’ were family members using online lending (OR = 5.527, 95% CI = 1.784–17.125) and lower scores on HA (OR = 0.938, 95% CI = 0.888–0.990). The risk factors for females’ online lending were family members using online lending (OR = 2.288, 95% CI = 1.201–4.362), hedonism (OR = 5.913, 95% CI = 1.327–26.341), and higher scores on mother’s punishment (OR = 1.099, 95% CI = 1.007–1.199).

**Conclusion:**

The utilization rate of online lending in males was significantly higher than in females. More attention should be paid to gender differences and the impact of family members’ using online lending on students when intervening in online lending.

## Introduction

Various kinds of online lending platforms are expanding as China’s Internet economy grows. Online lending in China currently has no official definition and is split into two categories: platforms for e-commerce services like Ant Check Later, and lending platforms for reducing financial stress, such as P2P, Ant Jie-bei (Chang, 2018). As its application procedure does not require the permission of students’ parents or legal guardians and its approval is fast (even in a few minutes), online lending overspreads quickly among college students. Despite the government’s efforts to crack down on P2P with high-interest rates, many students continue to use online lending, such as Ant Jie-bei. Thus, the shortcomings, influencing factors, and intervention strategies of online lending became the research foci for Chinese scholars.

Studies abroad on college students’ debt revealed that the debt in medical students increased yearly and had a negative effect on their choice of internship, marriage age, graduate destination, and quality of life (Jette, 2016; Ling et al., 2018; Gray et al., 2019; Amin et al., 2021). On the other hand, although the annual interest rate of online lending is above 13%, many college students (approximately 42%) still hold a positive attitude toward it. Additionally, in repayment, more than half of college students transferred the loans to their parents; worse still, some students repaid the debt by applying for another online lending (Xiao et al., 2018; Tang and Tang, 2020).

According to some scholars’ conclusions based on qualitative research, there were three critical psychological factors (conformity, comparison, and hedonism) and several intervening strategies for online lending, including conducting ideological and political education, strengthening legal supervision, and enhancing risk awareness (Dong, 2017; Xiao et al., 2018; Yang et al., 2019; Tang and Tang, 2020). The deficiencies of previous studies were the lack of quantitative research and gender differences in online lending. Thus, this study aims to analyze the gender differences in psychological risk factors and offer some practical suggestions for the intervention by quantitative methods.

## Materials and methods

### Study population and setting

This was a cross-sectional survey. A total of 984 questionnaires (from 349 males and 635 females) were obtained after excluding 316 invalid questionnaires with the artificial method by three prominent researchers, including the same choice for each question, two-thirds of options blank, or regular answers (effective rate = 75.69%). The questionnaire consisted of demographic information (Table 1), monthly expenses, self-evaluation for conformity, comparison, and hedonism (Table 2), as well as the following three psychological assessment instruments. Informed consent was secured from all participants.

**Table 1:** Descriptive statistics of participants’ demographic information.

Variable	n (N = 984)	%
Gender		
Male	349	35.47
Female	635	64.53
Parental education		
Primary school	243	24.7
Junior middle school	388	39.4
Junior high school	235	23.9
Junior college	76	7.7
Graduate and above	42	4.3
Family structure		
Single parent family	126	12.8
Core family	858	87.2
Using online lending		
Yes	681	68.2
No	303	30.8

**Table 2:** The survey questions of three central psychology causing online lending based on empiricism.

Questions	Answers
Conformity: People around are using online lending, I should follow this trend	0-disagree; 1-agree
Vanity: Luxury will attract more envious persons	0-disagree; 1-agree
Hedonism: We should enjoy life as soon as possible even through using online lending	0-disagree; 1-agree

### Assessment instruments

#### The Chinese version of SWLS for life satisfaction (C-SWLS)

The SWLS is widely used to measure life satisfaction and contains five items scored on a 7-point Likert scale (1, strongly disagree, to 7, strongly agree) (Diener et al., 1985). It has measurement invariance across gender and age and high internal reliability with *Cronbach’s α* > 0.70 (Bai et al., 2011; Silva et al., 2014).

#### The Chinese version of EMBU for parenting styles (C-EMBU)

The C-EMBU involves six subscales for the father’s parenting styles, including emotional warmth, punishment, over-interference, favoring subjects, rejection, and over-protection; five subscales for the mother’s parenting styles with emotional warmth, punishment, favoring subjects, rejection, and over-protection. The subjects evaluated 116 items by using a 4-point Likert scale (1-never, to 4- always). The *Cronbach’s α* for each subscale ranged from 0.46–0.88 (Yue et al., 1993).

#### The 144-item Chinese version of TCI for personality (TCI-144)

The TCI-144 consisted of a four-factor structure for temperament domains and a three-factor structure for character domains as follows: novelty seeking (NS), harm avoidance (HA), reward dependence (RD), persistence (P), self-directedness (SD), cooperativeness (C), and self-transcendence (ST). The subjects evaluated the items using a 5-point Likert scale (1, “very unlikely” to 5, “very likely”). The *Cronbach’s α* for each subscale ranged from 0.488 to 0.823 (Chen et al., 2013).

### Data analysis

Data analysis was conducted using IBM SPSS Statistics 19.0. Data interpolation was used to supplement the missing data. *χ*^2^-test/*t*-test was used to examine the gender differences among variables, and binary logistic regression was used to explore the risk factors of online lending for males and females, respectively. The level of statistical significance was set at *p* < 0.05.

## Results

### The gender differences among variables

A total of 681 students had used online lending (utilization rate = 68.2%). The comparisons for using online lending, monthly living expenses, the scores of SWLS, family parenting styles, and TCI-144 between genders are listed in Table 3. In terms of the utilization rate of online lending, negative parenting styles (punishment, over-interference, rejection, and over-protection in father’s parenting styles; punishment, rejection, over-protection in mother’s parenting styles), NS, C, and St, males were significantly higher than females. In contrast, positive parenting styles (emotional warmth of father’s parenting style and mother’s parenting style), HA, RD, and SD in males were significantly lower than that of females.

**Table 3:** Comparisons of variables between different genders.

Variable	Gender (N/M ± SD)		*T*/*χ*^2^	*P*
	Male (*N* = 349)	Female (*N* = 635)		
Using online lending				
Yes	269	412	15.718	0.000
No	80	223		
Monthly expenses (Yuan)	1333.7.41 ± 410.89	1324.91 ± 348.03	0.356	0.722
SWLS	22.63 ± 6.50	23.73 ± 6.21	−2.588	0.009
Father’s parenting styles				
Emotional warmth	51.98 ± 9.99	54.43 ± 11.10	−3.543	0.000
Punishment	20.18 ± 6.35	16.80 ± 5.03	8.566	0.000
Over-interference	20.02 ± 4.07	18.10 ± 4.08	7.067	0.000
Rejection	9.80 ± 3.11	8.72 ± 2.87	5.348	0.000
Over-protection	12.26 ± 2.74	11.36 ± 2.85	4.778	0.000
Favoring subjects	10.44 ± 3.21	10.63 ± 3.84	−0.857	0.392
Mother’s parenting styles				
Emotional warmth	50.23 ± 9.84	53.36 ± 10.84	−4.592	0.000
Punishment	14.52 ± 5.23	12.33 ± 3.93	6.858	0.000
Over-interference	33.26 ± 6.69	30.93 ± 6.97	5.087	0.000
Rejection	13.53 ± 5.03	12.02 ± 4.22	4.771	0.000
Favoring subjects	10.42 ± 3.37	10.49 ± 3.52	−0.307	0.759
Personality				
NS	58.17 ± 5.61	55.89 ± 6.33	5.828	0.000
HA	59.63 ± 6.47	61.48 ± 7.56	−4.029	0.000
RD	59.71 ± 5.01	61.36 ± 5.80	−4.673	0.000
*P*	61.66 ± 8.11	61.72 ± 8.06	−0.110	0.912
SD	61.35 ± 8.44	62.59 ± 9.14	−2.089	0.037
*C*	57.52 ± 7.04	54.00 ± 7.28	7.342	0.000
ST	56.07 ± 10.56	53.71 ± 9.51	3.581	0.000

### The risk factors of online lending for males and females

Binary logistic regression was used to explore the risk factors of online lending for males and females. Both of the models were well fitted (*χ*^2^_male_ = 7.362, *p* = 0.498, *Cox & Snell R*^2^ = 0.179; χ^2^_female_ = 9.155, *p* = 0.329, *Cox & Snell R^2^* = 0.107). Family members using online lending and low harm avoidance are the risk factors for males’ online lending (Table 4). The number of male users whose family members also used online lending was 5.527 times that of those whose family members did not. Each unit increase in the scores of injury avoidance was associated with a 0.062 times reduction in the risk of online lending. Family members using online lending, hedonism, and mother punishment are the risk factors for females’ online lending (Table 5). The number of female users whose family members also used online lending was 2.288 times that of those whose family members did not. The number of female users who were hedonistic was 5.931 times that of those who were not. Every one-unit increment of the scores of mother punishment was a 0.099 times increase in the risk of online lending. The most important explanatory variable was the family members using online lending in the males’ model (Wald = 8.780) and in the females’ model (Wald = 6.327).

**Table 4:** Binary logistic regression of online lending for males.

	Reference		*B*	S.E.	Wald	*P*	OR	95% CI
Family members using online lending	No	Yes	1.710	0.577	8.780	0.003	5.527	1.784–17.125
HA			−0.064	0.028	5.326	0.021	0.938	0.888–0.990

**Table 5:** Binary logistic regression of online lending for females.

	Reference		*B*	S.E.	Wald	*P*	OR	95% CI
Family members using online lending	No	Yes	0.828	0.329	6.327	0.012	2.288	1.201–4.362
Hedonism	Disagree	Agree	1.777	0.762	5.436	0.020	5.913	1.327–26.341
Punishment of mother’s parenting style			0.094	0.044	4.497	0.034	1.099	1.007–1.199

## Discussion

### Utilization rate of online lending

The utilization rate for online lending among medical students in Chengdu was 68.2%, much higher than the other studies based in different cities (Table 6; Zhang et al., 2018; Fu et al., 2019; Zhang, 2019; Pan, 2020; Li, 2021; Shi et al., 2021). Comparing the surveys conducted in Chengdu in 2018, it could be deduced that the utilization rate increases over time, which could be attributed to the widespread advertising of online lending platforms. Therefore, it is crucial to strengthen online lending platforms’ supervision actively. Additionally, a correlation study was conducted between the utilization rate of online lending and the annual GDP for each city by assuming that the utilization rate might have a relationship with the city’s GDP (Table 6). Sadly, the correlation coefficient was only 0.5 and was not statistically significant (*p* = 0.253). The reason might be that few quantitative studies can be studied for correlation analysis. As a result, our assumption should be explored by more quantitative research in the future.

**Table 6:** The information and results of previous studies in China.

Cities	Conducted time	An estimated Monthly Expense	Rate of online lending	Number of questionnaires	Comparison of utilization rate of online lending	GDP (Unit: Yuan)
Chengdu	2022	1,300	68.2	984	male>female	1.99 trillionin 2021
Dongguan	2021		33.89			1.09 trillion
Datong	2021	1,200	42.2	382		168.1 billion
Changchun	2020	1,250	84.18	411		663.8 billion
Changde	2019	1,200	5.8	241		362.42 billion
Zaozhuang	2019	1,200	6	396		169.39 billion
Chengdu	2018	1,000	50.6	1,118	Male > Female	1.57 trillion

### Gender differences in C-EMBU, TCI-144 and risk factors of online lending

Despite the fact that the Japanese version of EMBU differed from C-EMBU in some subscales, we found the following similarities: In contrast to females, males scored lower on the emotional warmth subscale and higher on the emotional rejection subscale (Someya et al., 2000). Besides, the parenting styles for males were more negative (rejection, punishment, and less communication) than that of females in our study. The reason might be that there are some parallels in child-rearing practices due to East Asian culture. However, it is crucial to note that the C-EMBU is different from the Japanese’s, and that there is little literature on gender differences in parenting styles among college students’ in China. Thus, more research needs to be done on our findings.

In line with other research in China and abroad, females in our study scored significantly higher on the HA than males. However, the findings for other subscales varied from different samples (Zohar et al., 2001; Chen et al., 2002; Shimizu and Ishikawa, 2011) and even were inconsistent with the research conducted in Beijing and the southwestern and northeastern regions of China, which also used TCI-144 as the assessment tool (Chen et al., 2013). Therefore, we speculate that the HA is mainly affected by biological factors, and the other subscales might be more related to sociocultural factors.

The results of the risk factors of online lending exhibited gender differences, which was in line with our hypothesis. The low score of HA was the risk factor for males only, and hedonism was solely for females (except for conformity and comparison). Thus, among many intervention measures based on qualitative research, our study showed that the effective ones were increasing education about the harm of online lending for males and ideological education about hedonism for females. Most notably, the variable of family members using online lending was the most important explanatory variable in the two models, consistent with data analyzed by neural networks. Furthermore, Bussmann and Giudici suggested that the Shapley-Lorenz-based approach was more accurate and explanatory than the above methods; therefore, it can be used in future risk management (Bussmann et al., 2020; Giudici and Raffinetti, 2021). To sum up, while intervening in students’ online lending, the community should actively intervene in their family members’ online lending.

## Conclusion

We should intervene in online lending rapidly because it is expanding quickly over time. Moreover, the impact of family members using online lending on students and gender differences should also be taken into consideration.

### Limitations

Here are some suggestions for future studies: first, our study’s generalizability was limited by the fact that it only included medical college students in Chengdu as a sample. We hope that the sample in future studies can be more diversified. In addition, the explanatory power of prediction variables to the utilization rate of online lending for males and females was 17.9 and 10.1%, respectively; thus, other risk factors (such as peer influence) need to be further studied.

## Data availability statement

The raw data supporting the conclusions of this article will be made available by the authors, without undue reservation.

## Ethics statement

The studies involving human participants were reviewed and approved by Chengdu Medical College. The patients/participants provided their written informed consent to participate in this study.

## Author contributions

All authors listed have made a substantial, direct, and intellectual contribution to the work and approved it for publication.

## Conflict of interest

The authors declare that the research was conducted in the absence of any commercial or financial relationships that could be construed as a potential conflict of interest.

## Publisher’s note

All claims expressed in this article are solely those of the authors and do not necessarily represent those of their affiliated organizations, or those of the publisher, the editors and the reviewers. Any product that may be evaluated in this article, or claim that may be made by its manufacturer, is not guaranteed or endorsed by the publisher.
